# Isolation and identification of a novel bacterium, *Pseudomonas* sp. ZyL-01, involved in the biodegradation of CL-20

**DOI:** 10.1186/s13568-020-01136-x

**Published:** 2020-10-31

**Authors:** Zhiyong Liu, Kai Dang, Cunzhi Li, Junhong Gao, Hong Wang, Yongchao Gao, Bin Zhao, Peng Fan, Airong Qian

**Affiliations:** 1 Lab for Bone Metabolism, Xi’an Key Laboratory of Special Medicine and Health Engineering, Key Lab for Space Biosciences and Biotechnology, Research Center for Special Medicine and Health Systems Engineering, NPU-UAB Joint Laboratory for Bone Metabolism, School of Life Sciences, Northwestern Polytechnical University, Xi’an, 710072 Shaanxi China; 2 Toxicology Research Center, Institute of Ordnance Industry Hygiene, Xi’an, 710065 Shaanxi China

**Keywords:** *Pseudomonas* sp. ZyL-01, Microbial community, Biodegradation, Explosive, Hexanitrohexaazaisowurtzitane

## Abstract

Hexanitrohexaazaisowurtzitane (CL-20) is a compound with a polycyclic cage and an N-nitro group that has been shown to play an unfavorable role in environmental fate, biosafety, and physical health. The aim of this study was to isolate the microbial community and to identify a single microbial strain that can degrade CL-20 with desirable efficiency. Metagenomic sequencing methods were performed to investigate the dynamic changes in the composition of the community diversity. The most varied genus among the microbial community was *Pseudomonas*, which increased from 1.46% to 44.63% during the period of incubation (MC0–MC4). Furthermore, the new strain was isolated and identified from the activated sludge by bacterial morphological and 16s rRNA sequencing analyses. The CL-20 concentrations decreased by 75.21 μg/mL and 74.02 μg/mL in 48 h by MC4 and *Pseudomonas* sp. ZyL-01, respectively. Moreover, ZyL-01 could decompose 98% CL-20 of the real effluent in 14 day’s incubation with the glucose as carbon source. Finally, a draft genome sequence was obtained to predict possible degrading enzymes involved in the biodegradation of CL-20. Specifically, 330 genes that are involved in energy production and conversion were annotated by Gene Ontology functional enrichment analysis, and some of these candidates may encode enzymes that are responsible for CL-20 degradation. In summary, our studies indicate that microbes might be a valuable biological resource for the treatment of environmental contamination caused by CL-20 and that *Pseudomonas* sp. ZyL-01 might be a promising candidate for eradicating CL-20 to achieve a more biosafe environment and improve public health.

## Key points

1. A new strain *Pseudomonas* sp. ZyL-01 capable of utilize CL-20 as the solely nitrogen source was isolated.
2. The reduction rate of *Pseudomonas* sp. ZyL-01 was performed in real effluent.
3. This is the first report about the biodegradation of CL-20 by microbial community.

## Introduction

Hexanitrohexaazaisowurtzitane (HNIW or CL-20), with the formula C_6_H_6_N_12_O_12_, is a nitroamine explosive that was first synthesized in 1987 (Nielsen et al. 1989). Due to its relatively superior explosive performance compared with conventional high-energy propellants, CL-20 has received extensive attention in recent years (Zhou et al. 2020). Therefore, it is highly anticipated to be a feasible and promising substitute for 2,4,6-trinitrotoluene (TNT), hexahydro-1,3,5-trinitro-1,3,5-triazine (RDX), and octahydro-1,3,5,7-tetranitro-1,3,5,7-tetrazocine (HMX). Nevertheless, there are still concerns regarding the environmental and health impacts from exposure as well as the harmful by-products of these explosives. For example, long-term exposure to TNT in water and soil potentially affects the development of *Daphnia similis* and the annelid *Enchytraeus crypticus* (potworm) (Kuperman et al. 2013; Ribeiro et al. 2012a, b). In addition, TNT and RDX are also toxic to mammals and have been regarded as dangerous chemical mutagens and potential human carcinogens (Chatterjee et al. 2017; Serrano-González et al. 2018). For instance, factory workers in Europe and the USA who were exposed to HMX have suffered several adverse effects to their nervous systems and livers (Won and Borden 2016). Meanwhile, sharing conserved similarities among their structures, CL-20 is also considered to have similar toxic effects as TNT, RDX, and HMX. It has been shown that extremely low concentrations of CL-20 in the soil even lead to a reduction in the density of earthworms (*Eisenia fetida*), suggesting that CL-20 appears to be more toxic to earthworms than TNT, RDX, and HMX (Panikov et al. 2007). Another study indicates that earthworm physiologies show clear retardation, stiffness, and body shrinkage after exposure to a low concentration of 0.02 μg/cm^2^ CL-20 compared with a high concentration of 0.21 μg/cm^2^ RDX (Gong et al. 2007). Additionally, CL-20 has been found to be toxic to avian species and mammals. For instance, in 14-day subacute and 42-day subchronic feeding experiments on the Japanese quail, embryos from birds exposed to CL-20 had multiple cranial and facial deformities, indicating that CL-20 may cause substantial defects in avian growth and development (Bardai et al. 2005). In addition, another study has shown that CL-20 has toxic effects in pregnant mice by teratogenic testing; simultaneously, it also has been demonstrated that CL-20 possibly causes chromosomal damage and aneuploidy formation by increasing the micronucleus rate (Wenxia et al. 2007). Therefore, an effective and environmentally-friendly strategy is required to decontaminate environments due to an increasing awareness of the potential harmful effects caused by CL-20.

To date, several biotic and abiotic reactions have been shown to be capable of degrading CL-20. For instance, CL-20 can be decomposed by alkaline hydrolysis and photodegradation (Balakrishnan et al. 2003; Hawari et al. 2004). However, the shortcomings of abiotic remediation technologies usually involve high costs and low efficiencies, which further limit their application in the real-world. Therefore, increasing attention has been eventually transferred to biological strategies, especially the microbial degradation of CL-20 (Bhushan et al. 2005a; Crocker et al. 2006; Karakaya et al. 2009; Trott et al. 2003). For example, both *Pseudomonas* sp. FA1 and *Agrobacterium* sp. JS71, isolated from soil, can grow properly using CL-20 as the sole nitrogen source (Bhushan et al. 2003; Trott et al. 2003). One surprising underlying mechanism behind its growth is that *Pseudomonas* sp. FA1 produces a flavoenzyme to decompose CL-20 at a rate of 3.2 nmol/h under anaerobic conditions. Likewise, another obligate anaerobic bacteria isolated from marine sediment, *Clostridium* sp. strain EDB2, secretes an NADH-dependent dehydrogenase, which also digests CL-20 at a rate of 2.6 nmol/h (Bhushan et al. 2004c, 2005a, b). Moreover, *Clostridium* sp. strain EDB2 has been shown to decompose RDX, HMX, and TNT (Bhushan et al. 2004c), the three most common cyclic nitramine explosives. Although several strains have shown the potential or capacity to decompose CL-20 under aerobic or anaerobic conditions, the inefficient degradation rates largely limit their practical applications. Therefore, additional investigations and the isolation of new possible strains with a stronger capacity and higher efficiency of degrading CL-20 are required. Importantly, utilizing microbial communities as biodegraders has many benefits, such as an environmentally-friendly efficiency, better environmental adaptability, and longer dynamic regulation when compared with conventional single strains. However, currently, there are no reports investigating the biotic degradation of CL-20 by a microbial community.

In this study, five microbial community samples were successfully acquired by successive microbial enrichment cultures. The dominant bacterial genera capable of degrading CL-20 were determined by monitoring the dynamic changes in their microbial compositions. Interestingly, a single strain was isolated and identified from the microbial community when CL-20 was used as the sole nitrogen source. The bacterium was named as *Pseudomonas* sp. ZyL-01 after morphological identification and 16s rRNA gene sequencing. Furthermore, the characteristics of the growth rate and the CL-20 degradation efficiency of *Pseudomonas* sp. ZyL-01 were also systematically determined. A final draft genome sequence of *Pseudomonas* sp. ZyL-01 was obtained and analyzed, thus providing valuable information regarding the potential biological functions of microbial communities. The data of the CL-20 biodegradation abilities of microbial communities and the newly isolated strain *Pseudomonas* sp. ZyL-01 will enable us to gain further insights into the possible microbial degradation mechanisms of CL-20. Additionally, all findings may provide valuable information for designing biotechnological tools and strategies for CL-20 decontamination.

## Materials and methods

### Materials and CL-20 reagents

Activated sludge was collected from the sewage treatment plants of a CL-20 manufacturing factory (Liaoyang City, Liaoning Province, China). The CL-20 samples, with a purity greater than 99%, were obtained from Xi'an Modern Chemistry Research Institute. All other reagents were of analytical grade or chemically purified.

### Culture of microbial community and 16s rRNA amplification

The 5 g activated sludge samples were added to a 100-mL conical flask containing 25 mL of sterile Luria–Bertani (LB) medium (10 g/L tryptone, 5 g/L yeast extract, and 10 g/L NaCl, autoclaved at 121 ℃ for 20 min). After incubation in a shaker at 37 ℃ and 120 rpm for 24 h, 1 mL of the culture was taken and transferred to 25 mL of fresh mineral salt medium (MSM) (3.8 g/L Na_2_HPO_4_·12H_2_O, 1.5 g/L KH_2_PO_4_, 10 mL of 0.755 g/L CaCl_2_; 10 mL of filtered sterilized mixed solution: 5.0 g/L MgSO_4_·7H_2_O, 0.152 g/L MnSO_4_·H_2_O, 0.5 g/L FeSO_4_·7H_2_O, and 20% glucose as the carbon source). All reagents mentioned above were autoclaved before use, if not specified otherwise. CL-20 acetone solution (10 g/L) was added to the MSM so that the solution contained 100 mg/L CL-20. The culture was incubated at 28 ℃ and 120 rpm for 7 days. After five successive transfers, five microbial community samples (named MC0 to MC4) were acquired. The microbial composition of each sample was determined using 16s rRNA-based metagenomics analysis. Briefly, a microbial community sample (1 mL) was centrifuged at 12,000 rpm for 5 min, followed by discarding the supernatant. The centrifugation process was repeated three times. DNA extraction was performed using an E.Z.N.A™ Mag-Bind Soil DNA Kit (OMEGA), and the DNA integrity was checked by agarose gel electrophoresis. Polymerase chain reaction (PCR) amplification was carried out using 16s rRNA primers (F: 5ʹ-CCTACGGGNGGCWGCAG-3ʹ; R: 5ʹ-GACTACHVGGGTATCTAATCC-3ʹ), and the PCR product was purified and subsequently sent to Sangon Biotech Co., Ltd. (Shanghai, China) for metagenomic sequencing.

### Bioinformatics analysis of microbial communities

To obtain high-quality datasets, Flash v1.2.3 and Qiime v1.8.0 software were used for sequence splicing, filtering, and processing of clean readouts of the original data. Usearch v5.2.236 software was utilized to calculate operation classification unit (OTU) clustering based on the similarity score. RDP classifier 2.12 was used for annotation of species of representative OTU sequences, and the sequence alignment was performed by the basic local alignment search tool (BLAST). The alpha-diversity, which represents the richness and diversity of the microbial community, was also determined (Bissett and Brown 2018; Wang et al. 2019).

### Isolation and identification of the bacterial strain

The microbial community sample was inoculated onto an MSM agar plate containing 100 mg/L CL-20 as the sole nitrogen source. A single colony was picked out and transferred to a liquid MSM culture after incubation for 48 h, followed by shaking at 28 ℃ and 120 rpm for another 48 h. After 4–5 successive rounds of incubation, the bacteria were purified and identified by the morphological characteristics of the colony. A 1-mL aliquot of bacteria was centrifuged at 12,000 rpm for 5 min, the supernatant was discarded, and the bacterial pellet was suspended and separately stored in LB medium and MSM at − 80 ℃. The identified strain *Pseudomonas* sp. ZyL-01 (Preservation number CGMCC No. 18373) was deposited in the China General Microbiological Culture Collection Center (CGMCC).

Bacteria in the logarithmic phase of growth were streaked and inoculated into 25 mL of MSM, cultured at 28 ℃ for 48 h, and observed for colony shape, color, viscosity, ridge, and edge morphology. A single colony was selected for Gram staining, followed by observation of the size, shape, flagella, and Gram reaction under a microscope. The isolated strain was identified by 16s rRNA gene sequencing analysis. The obtained sequences were compared with the existing 16s rRNA gene sequences in GenBank by BLAST. The phylogenetic tree was built by the neighbor joining tree method using MEGA-X-10.0.5 software (Kumar et al. 2018).

### Growth characteristics of the bacteria

A single colony from a solid culture plate was inoculated into 25 mL of LB culture medium and cultured in a shaker at 28 ℃ and 120 rpm until the bacteria reached the logarithmic growth phase. A 25-µL aliquot of culture was then inoculated into 25 mL of fresh LB culture medium and then grown at different temperatures (21 ℃, 28 ℃, and 37 ℃) and pH values (4.0, 5.5, 7.0, 8.5, and 10.0), and the microbial growth of each sample was determined by measuring the optical density at 600 nm (OD_600_). All the tests were performed in triplicate and repeated three times.

### Detection of CL-20 degradation by bacteria

The LB culture at the logarithmic phase of growth (500 μL) was centrifuged at 12,000 rpm for 5 min, followed by removal of the supernatant. The bacteria were resuspended and washed twice in 1 mL of sterile water and then transferred to a vial with 5 mL of liquid MSM containing CL-20 as the sole nitrogen source. The culture was incubated on a shaker at 28 ℃ and 120 rpm; meanwhile, the concentration of CL-20 was determined by high-performance liquid chromatography (HPLC) at different time points. The procedures were performed as described previously (Monteil-Rivera et al. 2004). In brief, a vial of MSM containing culture was left in the fume hood for evaporation overnight, followed by extraction with 10 mL of acetonitrile and ultrasonic treatment for 4 h at 20 ℃ in the dark. After centrifugation at 4500 rpm for 30 min, a 5-mL sample of supernatant was added to 5 mL of CaCl_2_-NaHSO_4_ solution at two different concentrations (5 g/L and 0.2 g/L). The samples were then shaken and left for another 30 min, filtered through a 0.22-µm Millipore filter, and analyzed by HPLC (Waters^®^ e2695 Separations Module, Waters Corp., Milford, MA, USA). The separation was completed on a CORTECS C18 column maintained at 30 ℃. The mobile phase (70% aqueous methanol) was run at 1 mL/min for 8 min. The detector was set to scan from 200 to 350 nm. Chromatograms were extracted at 230 nm, and the injection volume was 20 μL. All the experiments were performed in triplicate and repeated three times.

### Biodegradation assay in effluent by ZyL-01

The effluent sample was collected from the crystal transfer process of CL-20 plant, which contained chloroform (0.374 g/L), ethyl acetate (1.235 g/L) and CL-20 (42.32 mg/L). The bacteria from LB culture (500 μL) at the logarithmic phase of growth was centrifuged and washed for twice, and then transferred to the following 50 mL filtered sterilized effluent samples: (a) effluent sample with bacteria; (b) effluent sample with glucose (2 g/L) as a carbon source and bacteria; (c) effluent sample without bacteria and any supplement as abiotic control. The samples were incubated at 28 ℃ and 120 rpm for 16 days, and the concentration of CL-20 was determined by HPLC as mentioned above. All the experiments were performed in triplicate.

### Bioinformatics of the draft genome sequence of *Pseudomonas* ZyL-01

The sequencing of the selected *Pseudomonas* sp. ZyL-01 was performed by Sangon Biotech Co., Ltd. (Shanghai, China). The DNA library of *Pseudomonas* ZyL-01 was constructed with purification by a NEB NextR Ultra™ DNA Library Prep Kit and conventional PCR amplification. The length distribution of the library was determined by an Agilent Technologies 2100 DNA 1000 Kit and used for quality control. The purified DNA pool was sequenced by Illumina HiSeq. The sequencing reads were cleaned up using Trimmomatic and assembled by SPAdes, and then the contig GAP was filled by GapFiller and one scaffold was obtained for further analysis. Finally, the coding genes were predicted using Prokka, while the tRNAs and rRNAs were identified by Aragorn and RNAmmer. The potential functions of the coding genes were predicted using the databases of Swiss-Prot, Cluster of Orthologous Groups of proteins (COGs), Gene Ontology (GO), Kyoto Encyclopedia of Genes and Genomes (KEGG), NCBI nucleotide sequences (NT), NCBI nonredundant protein sequences (NR), and Conserved Domain Database (CDD).

## Results

### Dynamic compositional changes of microbial communities

As shown in Table 1, the average reads of the five microbial communities (MC0–MC4) was 75,359 and the Good’s coverage of MC4 reached 0.98, indicating that the information obtained was sufficient to reveal most members of the microbial community. Regarding the enrichment of the samples, the OTU numbers decreased from 10,572 (MC0) to 1363 (MC4), the alpha indexes including Shannon, ACE, and Chao1 showed a decreasing tendency, and the Simpson index increased, which all suggest that the enrichment and diversity of the community declined substantially.

**Table 1:** OTU numbers and diversity index results of the microbial communities

Sample	Read no.	OTU no	Shannon index	ACE index	Chao1 index	Coverage	Simpson
MC0	58,989	10,572	6.37	170,264.84	67,057.38	0.85	0.02
MC1	94,667	9781	4.35	133,303.40	55,530.89	0.92	0.13
MC2	87,687	10,378	4.62	165,549.65	65,450.23	0.90	0.08
MC3	57,315	2705	2.70	115,128.33	35,685.73	0.96	0.21
MC4	78,137	1363	2.57	44,476.73	18,290.5	0.98	0.15

**Fig. 1:**
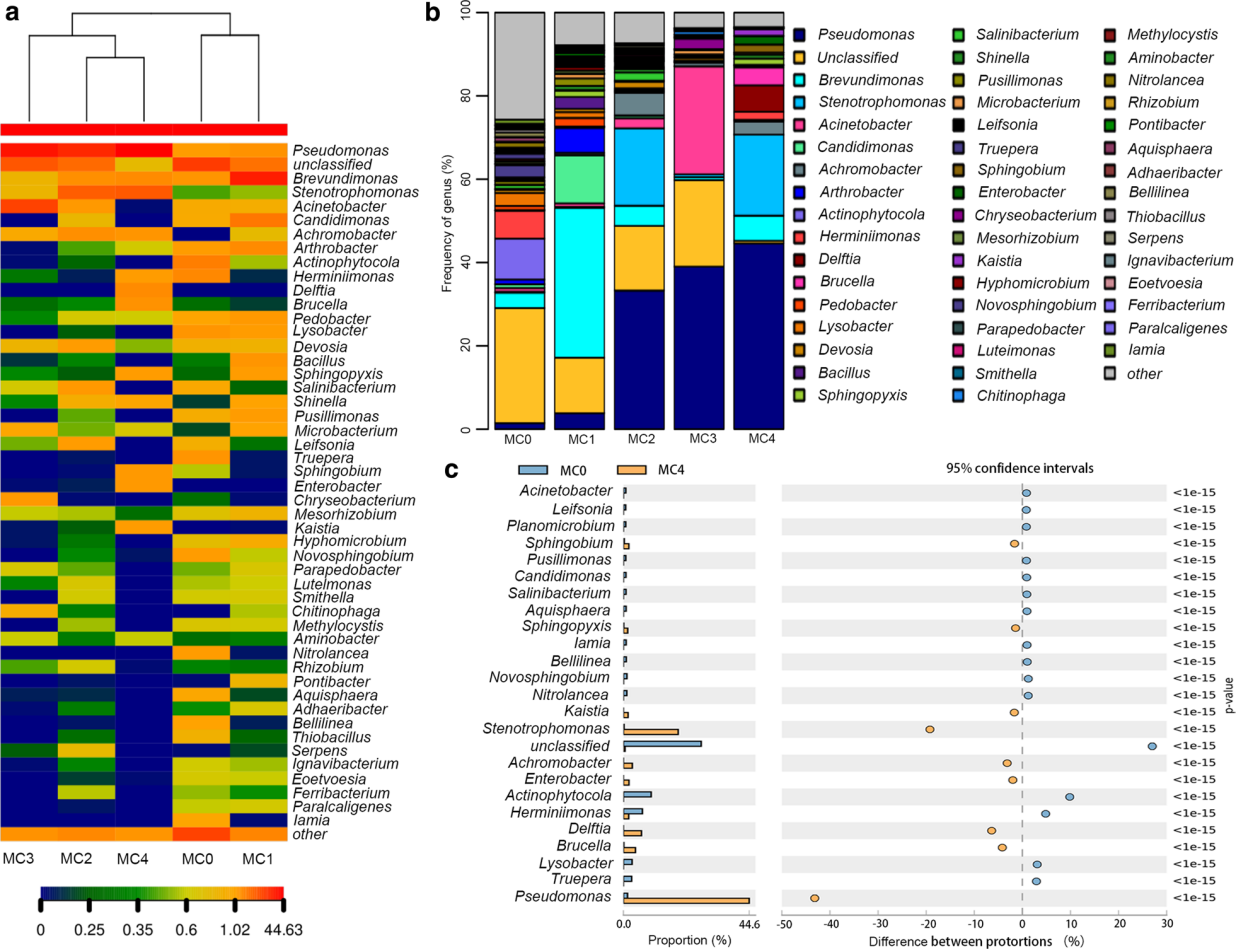
Dynamic composition changes of microbial communities, heatmap of genus (**a**), microbial communities composition changes (**b**), genus difference between MC0 and MC4 (**c**)

A total of 623 genera were detected in MC0, while only 151 genera were left in MC4 after five rounds of transfer. The frequency change of the genera and the structures of the group in the bacterial community among the samples were explored. As shown in the heat map of the microbial community (Fig. 1a), three clusters were continuously found in all five samples. MC0 and MC1 were classified into the first cluster, whereas MC2 and MC4 were classified into the second cluster. In particular, MC3 alone comprised the third cluster. As shown in Fig. 1b, the 16s rRNA read numbers showed that the predominant bacterial genus were *unclassified* (27.60%) in MC0 and *Brevundimonas* (35.96%) in MC1, in which *Pseudomonas* genus was less abundant, and the ranking was 7th in MC0 and 5th in MC1. Interestingly, *Pseudomonas* sp. gradually became the dominant bacteria during the culturing process, which accounted for 33.28% (MC2), 39.02% (MC3) and 44.63% (MC4), respectively. In fact, a dramatic increasing has been found with the proportion of *Pseudomonas* sp. between MC0 and MC4 (Fig. 1c), indicating that it may play a key role in the biodegradation of CL-20.

### Characterization of the isolated bacterium

**Fig. 2:**
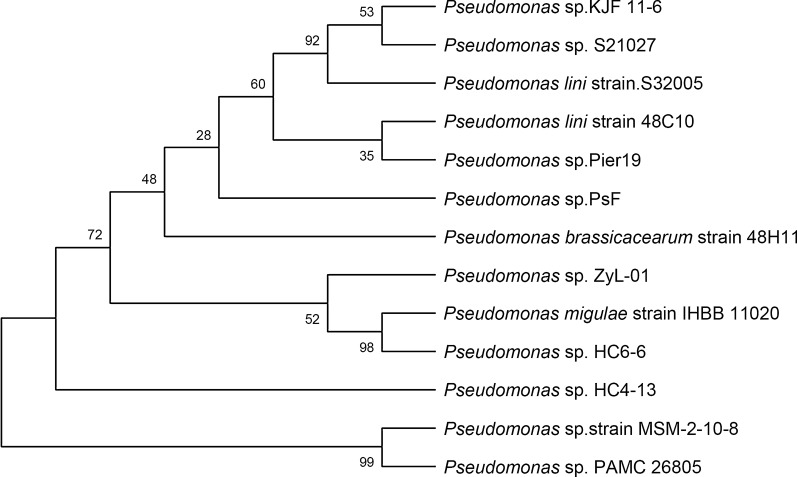
Phylogenetic tree of the newly isolated strain ZyL-01

In our study, a strain capable of utilizing CL-20 as the sole nitrogen source was identified and named as *Pseudomonas* sp. ZyL-01. By morphological observation, the colony was white, had a round shape with a diameter of about 1–2 mm, and the surface of the colony was wet and easy distorted (Additional file 1: Fig. S1). Microscopic observation showed that the bacteria were rod-shaped, 2–3 μm in length, flagellum-free, and Gram negative. The length of the 16s rRNA of the ZyL-01 strain was 1479 bp (Additional file 2: Fig. S2), sharing 99% similarity with *Pseudomonas* migulae strain IHBB 11020 and *Pseudomonas* sp. HC6-6, and the three bacteria were clustered within the same subbranch in the phylogenetic tree (Fig. 2), indicating that the strain belongs to *Pseudomonas* strains.

The growth characteristics of ZyL-01 were monitored by measuring the OD_600_ under different temperatures and pH values. At 21 ℃, the bacteria began to grow at 12 h, and the logarithmic growth period was from 16 to 30 h, followed by a stable growth phase from 30 h. However, when the incubation temperature changed to 28 ℃, the logarithmic growth period started earlier, from 14 to 24 h, even though the starting point of growth remained the same at 12 h. Moreover, at 37 ℃, *Pseudomonas* sp. ZyL-01 started to grow at 14 h, with a logarithmic growth period from 16 to 26 h (Additional file 3: Fig. S3a). The growth characteristics of *Pseudomonas* sp. ZyL-01 were also monitored under different pH values at 28 ℃. The OD_600_ increased to 0.326 and 0.521 at a pH of 5.5 and 7.0, respectively; whereas the bacteria did not grow at other pH values (Additional file 3: Fig. S3b). Combined with the previous data, all of these results suggest that the strain was able to grow at temperatures between 21 and 37 ℃ and pH values between 5.5 and 8.5, with the possible optimal temperature of 28 ℃ and pH of 7.0 (Additional file 3: Fig. S3b).

### CL-20 biodegradation assay by ZyL-01 and microbial community

**Fig. 3:**
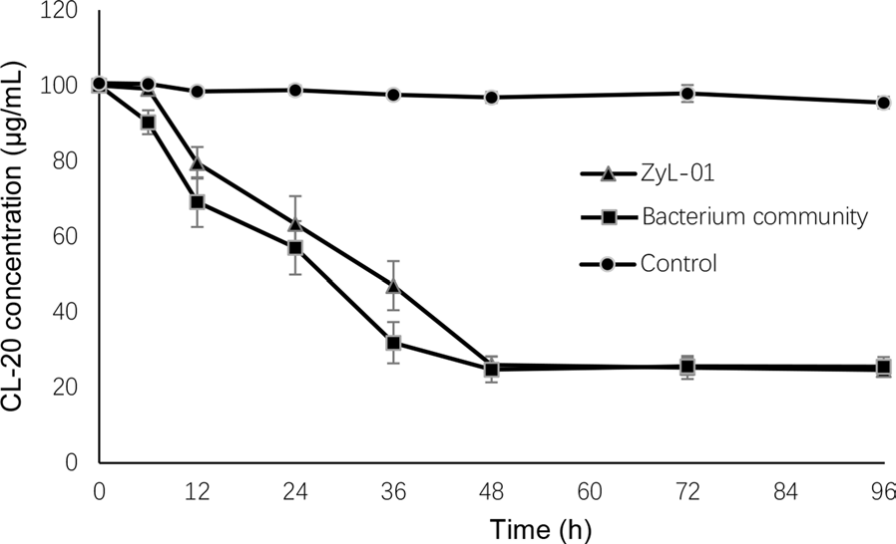
Biodegradation of CL-20 by ZyL-01 and the MC4

To compare the biodegradation of CL-20 by *Pseudomonas* sp. ZyL-01 and MC4, another culture study was performed. The concentration of CL-20 (100 μg/mL) started to decrease by ZyL-01 after incubation for 6 h, while 74.02 μg/mL CL-20 was degraded within 48 h when CL-20 was the sole nitrogen source (Fig. 3). By contrast, the concentration of CL-20 decreased by the bacterial community starting from 0 h, demonstrating that the initial decomposition rate of the bacterial community was slightly faster than that of the single colony (Fig. 3). After 48 h, the bacterial community degraded 75.21 μg/mL CL-20. Strikingly, the final amount of degraded CL-20 was approximately equal to that by ZyL-01. In the abiotic control group, CL-20 only decreased by 4.54 μg/mL in 96 h. Altogether, these data suggest that the decrease of CL-20 was largely dependent on the microbial activity.

### Biodegradation of CL-20 in production effluent by ZyL-01

**Fig. 4:**
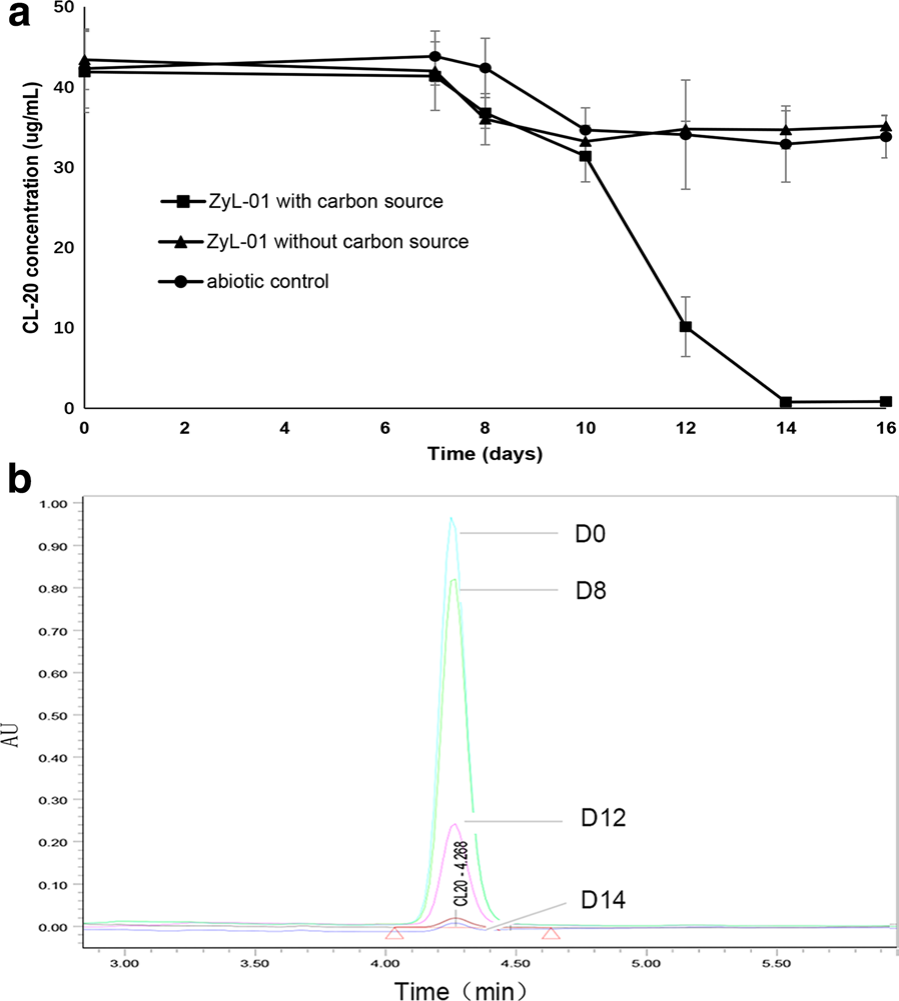
Biodegradation of CL-20 in production effluent by ZyL-01, CL-20 concentration changing in three groups (**a**), CL-20 Liquid chromatograms in ZyL-01 with carbon source group (**b**)

The effect of CL-20 biodegradation in real effluent by ZyL-01 was shown in Fig. 4. Within 7 days incubation, change of CL-20 concentration was not found in the three groups. There was a slight decrease of CL-20 during 8th to 10th days. Subsequently, there was significantly decrease of CL-20 in the ZyL-01 with carbon source group in 12th day, compared with the abiotic control and ZyL-01 without carbon source groups (Fig. 4a), indicating the target bacteria could degrade CL-20 in the presence of carbon source. Ultimately, ZyL-01 decomposed 98% CL-20 of production effluent in 14 days’ incubation, and the concentration of CL-20 decreased from (41.90 ± 5.06) μg/mL to (0.78 ± 0.20) μg/mL (Fig. 4b).

### Draft genome sequence of *Pseudomonas* sp. ZyL-01

The length of the *Pseudomonas* ZyL-01 genome was 6,995,221 bp, with 41.12% GC content (Table 2). A total of 6491 protein-coding genes were annotated in the *Pseudomonas* sp. ZyL-01 genome, with an average length of 950.11 bp and a coding ratio of 88.16% (Table 2). Moreover, a total of 69 tRNAs and 4 rRNAs were identified (Table 2). A total of 99.36% of the unigenes were annotated by at least one database, whereas 41.98% of the unigenes were annotated if all eight databases were used. Apart from KEGG (44.09%), more than 70% of the sequences can be allocated into biological functions using one of the other databases. In particular, TrEMBL was the database in which most of the unigenes (99.21%) were annotated (Additional file 4: Table S1).

**Table 2:** Genomic features of *Pseudomonas* ZyL-01

Feature	Value
Genome length (bp)	6,995,221
GC content (%)	59.48
Protein coding genes	6491
Min length (base)	49
Max length (base)	12,984
Average length (base)	950.11
Total coding genes (base)	6,167,147
Coding ratio (%)	88.16
tRNA	69
rRNA	4

**Fig. 5:**
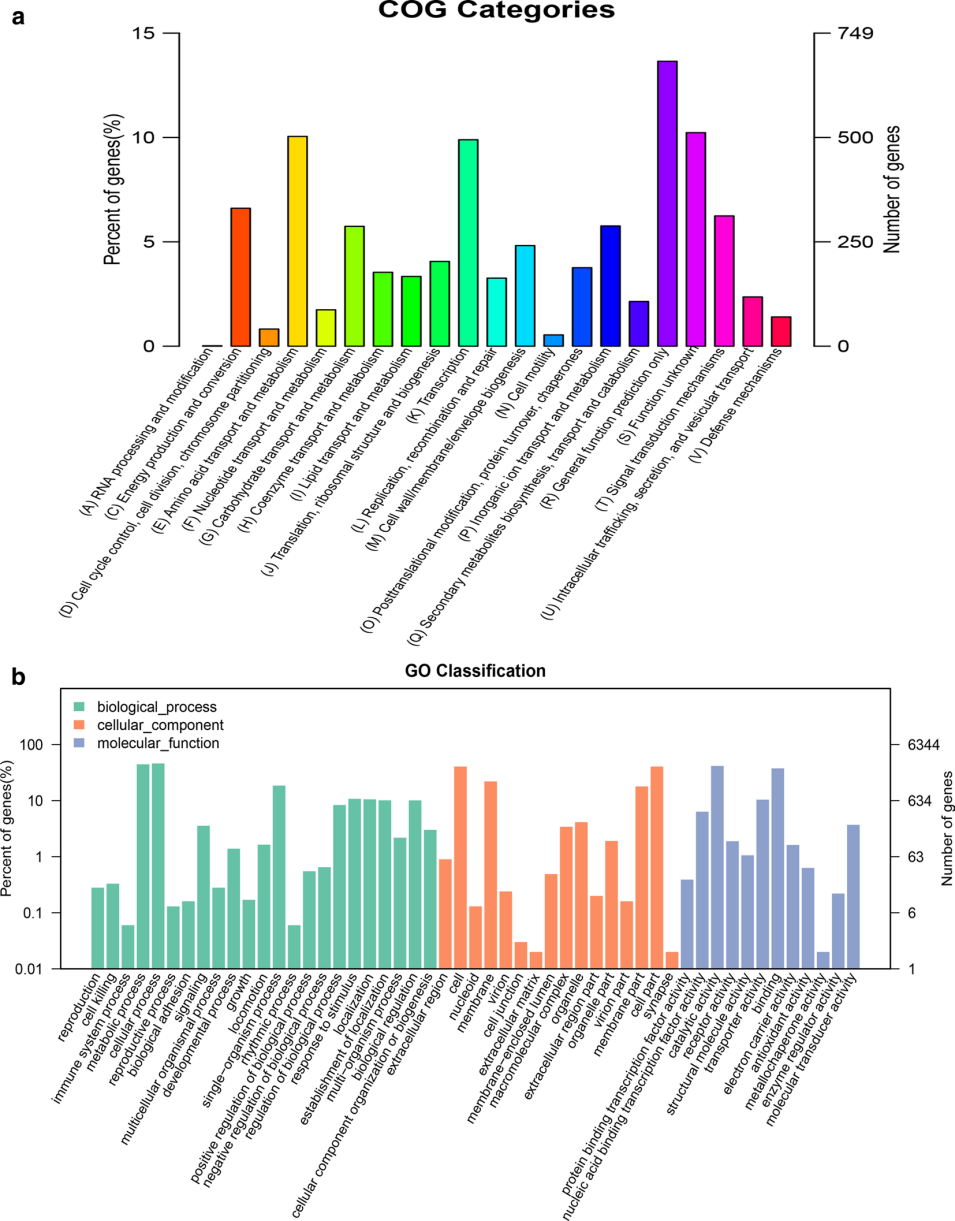
Unigene annotation in COG (**a**) and GO (**b**)

The main feature of *Pseudomonas* sp. ZyL-01 found in this study was to degrade CL-20. According to COG analysis in one study (Jia et al. 2020), 330 genes were mapped to the energy production and conversion category (Fig. 5a), which may include genes encoding the enzymes that can degrade CL-20. For instance, four nitroreductases (PROKKA_02307, PROKKA_04681, PROKKA_04840, and PROKKA_04929) were annotated by COG (Sree et al. 2019) and were recognized as being involved in oxidoreductase activity by GO (Fig. 5b).

## Discussion

In this study, a microbial community and a new strain (*Pseudomonas* ZyL-01) capable of utilizing CL-20 as the sole nitrogen source were acquired and identified from the activated sludge. To the best of our knowledge, this is the first report investigating the biodegradation of CL-20 by a microbial community. The present study demonstrated that the decontamination rate of CL-20 by *Pseudomonas* sp. ZyL-01 was much higher than that by previously reported strains. Furthermore, the effect of CL-20 biodegradation in the real effluent was conducted. Therefore, we considered that *Pseudomonas* ZyL-01 can offer an effective solution for better degradation of CL-20 in contaminated environments.

High-throughput sequencing technology has been successful employed in studying the whole-genome diversity of microbial communities in complex environments (Nahidul-Islam et al. 2018; Wang et al. 2020). To better understand the dynamic changes of the microbial community, 16s rRNA metagenomic high-throughput sequencing technology was performed (Alisawi et al. 2017). As shown in Table 1, the Chao estimator, Ace estimator, Shannon index, and Simpson index results all demonstrate a clear declining tendency in richness and diversity along with time. Consistently, a similar decrease in microbial community diversity during the enrichment culture process also has been observed in another study (Wang et al. 2017). As shown in Fig. 1, the composition of our samples changed dramatically, and the largest proportional change among them was *Pseudomonas* strain, which increased from 1.46% (MC0) to 44.63% (MC4). Several studies have shown that the microbial community plays vital roles in the degradation of nitro-aromatic explosives, such as TNT and RDX (Khan et al. 2015a, b); however, no information has been reported regarding the microbial degradation of CL-20. In our study, although the speed of CL-20 degradation by MC4 was almost equal to that by *Pseudomonas* sp. ZyL-01, Fig. 3 illustrated the initial speed of CL-20 degradation in response to MC4 was quicker than that of *Pseudomonas* sp. ZyL-01, indicating that the microbial community, overall, has an advantage over the single strain in the degradation of explosives, which also has been indicated in previous research (Robertson and Jjemba 2005). The reason for this phenomenon might be due to the effect not only by *Pseudomonas* sp. ZyL-01 but also other unknown bacteria.

Additionally, a new strain that utilizes CL-20 as the sole nitrogen source in MSM culture was successfully isolated and was eventually identified as *Pseudomonas* sp. by morphology and 16s rRNA gene sequencing (Fig. 2). Interestingly, the transformation of CL-20 by *Pseudomonas* strain has been described previously (Bhushan et al. 2003). *Pseudomonas* sp. FA1 can transform CL-20 under both aerobic and anaerobic conditions, with a maximum degradation rate of 10.95 μg/mL in 24 h (Bhushan et al. 2003). In contrast, the degradation rate of CL-20 by *Pseudomonas* sp. ZyL-01 was 74.02 μg/mL in 48 h (Fig. 3), which is much faster than that of the *Pseudomonas* sp. strain FA1. Moreover, a CL-20 degrading assay was conducted in the real production effluent, and the decreasing rate reached to 98% in the presence of glucose as supplement carbon source (Fig. 4), which further manifests the good performance of ZyL-01 in CL-20 decontamination. Nevertheless, the initial biodegrading time in real waste is much slower than in laboratory conditions, which is likely due to the complex ingredient including chloroform, ethyl acetate and so on. According to a previous report, chloroform is toxic to prokaryotes (Weathers and Parkin 2000). In addition to the degrading abilities of the biotic groups, a small reduction of 4.54 μg/mL in CL-20 biodegrading assay (Fig. 3) and 8.50 μg/mL in the real production effluent biodegrading assay (Fig. 4a) was also found in 96 h and 16 days respectively, which was probably due to alkaline hydrolysis (Balakrishnan et al. 2003). Another microorganism, *Agrobacterium* sp. strain JS71, has been reported to transform CL-20 (Trott et al. 2003); the same observation was also found for *Clostridium* sp. strain EDB2 (Bhushan et al. 2005a) and *Phanerochaete chrysosporium* (white-rot fungi) (Fournier et al. 2006). However, these strains mentioned above have a limited practical application largely due to their low degradation rates. Therefore, *Pseudomonas* sp. ZyL-01 found in this study is expected to perform a more fundamental role in environmental decontamination strategies.

More importantly, to better understand the possible mechanisms behind microbial CL-20 degradation, the draft genome sequence of *Pseudomonas* sp. ZyL-01 was obtained in the current study. In addition, 330 genes, annotated as protein-coding genes in several public databases, were recognized as being involved in energy production and conversion (Fig. 5); these genes may include potential candidates encoding the functional enzymes that are responsible for degrading CL-20. Previous studies (Bhushan et al. 2003, 2004c, 2005a) have shown that the degrading enzymes for nitromine chemicals are mostly NAD(P)H-dependent, as electron donation is required to transform the chemicals. For instance, nitroreductase, a member of the oxidoreductase enzyme family, can effectively degrade nitroaromatic compounds by transferring two electrons from NADPH to amines by reducing the nitro groups (Sree et al. 2019; Xu and Zhou 2017). Interestingly, as shown in Fig. 5, four other nitroreductase genes were annotated in our study, and these genes are likely involved in CL-20 degradation. It has been shown by computational density functional theory prediction analysis that homolytic NO_2_ elimination is the most favorable process in the initial stage of CL-20 unimolecular decomposition (Okovytyy et al. 2005). To the best of our knowledge, three signaling pathways, including denitration, nitroreduction, and denitrohydrogenation, have been proposed as being involved in the biodegradation of CL-20. For instance, nitroreductase (*E. coli*) (Bhushan et al. 2004a), flavin adenine dinucleotide (FAD)-containing salicylate 1-monooxygenase (*Pseudomonas* sp. strain ATCC 29352) (Bhushan et al. 2004b), and manganese peroxidase (*Phanerochaete chrysosporium*) (Fournier et al. 2006) catalyze two single-electron transfer reactions to conduct two sequential N-denitration reactions, thus further decomposing CL-20 into glyoxal and formic acid. Moreover, an NADH-dependent dehydrogenase secreted by *Clostridium* sp. strain EDB2 is also capable of degrading CL-20 via the three routes mentioned above (Bhushan et al. 2004c, 2005a, b), which are related to the initial transformation prior to ring cleavage. Therefore, the enzymes responsible for CL-20 degradation are likely NAD(P)H dependent as they provide a supply of donor electron(s). In our study, four genes in *Pseudomonas* sp. ZyL-01 (PROKKA_02307, PROKKA_04681, PROKKA_04840, and PROKKA_04929), which can encode FMN- or FAD-dependent and NAD(P)H-dependent enzymes, are able to metabolize nitro-substituted compounds and should be considered as candidate genes capable of degrading CL-20. Furthermore, the exact mechanisms by which the microbial community and *Pseudomonas* sp. ZyL-01 degrade CL-20 should be explored in more detail in future research.

In summary, a comprehensive microbial community from activated sludge was obtained in the present study by enriching cultures, among which 44.63% of the microbes belonged to the *Pseudomonas* genus, and proved to substantially reduce the concentration of CL-20 in the medium. In addition, a newly isolated CL-20-degrading strain (*Pseudomonas* sp. ZyL-01) was identified as *Pseudomonas* strain by morphological and 16s rRNA sequencing analyses. The microbial community and this strain can utilize CL-20 as the sole nitrogen source to grow, and their degradation efficiencies were investigated both in laboratory and real effluent conditions, which were much higher than those of previously reported strains. The protein-coding genes of the bacterium and their putative annotations were obtained by draft genome sequencing, thus providing valuable information for further exploration of the underlying mechanisms about how *Pseudomonas* sp. degrades CL-20. Most importantly, the microbial community and *Pseudomonas* sp. ZyL-01 found in this study are effective and promising alternative agents for eradication of CL-20 contaminants in nature.

## Supplementary information

**Additional file 1: Fig S1.** Isolation of a novel strain in an MSM agar plate (a), and for Gram staining (b).

**Additional file 2: Fig S2.** 16s rRNA gene sequencing analysis of the newly isolated strain ZyL-01.

**Additional file 3: Fig S3.** Growth characteristic of ZyL-01 with different temperatures (a) and pH values (b).

**Additional file 4: Table S1.** Numbers and percentages of unigenes in different databases.

## Data Availability

The genome sequencing raw data of the five microbial communities have been submitted to the Sequence Read Archive (SRA) database (https://submit.ncbi.nlm.nih.gov/subs/sra/SUB7594878) in the NCBI (Accession number: PRJNA639690). The sequencing raw data of the Pseudomonas sp. ZyL-01 genome has been deposited to SRA database (https://submit.ncbi.nlm.nih.gov/subs/sra/SUB7607413) under the following accession number: PRJNA640219.
