# Supplementary figures and images for: Isolation and identification of a novel bacterium, Pseudomonas sp. ZyL-01, involved in the biodegradation of CL-20
Source: AMB Express. 2020 Oct 31;10:196. doi: 10.1186/s13568-020-01136-x (PMC7603440; doi:10.1186/s13568-020-01136-x)

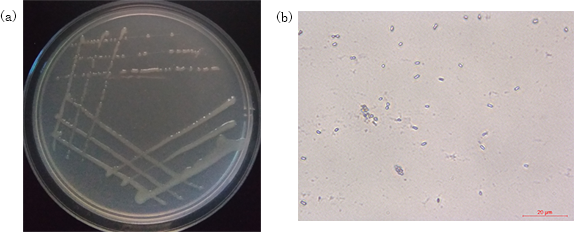

Supplement: Supplementary file 1 — Additional file 1: Fig S1. Isolation of a novel strain in an MSM agar plate (a), and for Gram staining (b). [file 13568_2020_1136_MOESM1_ESM.tif]

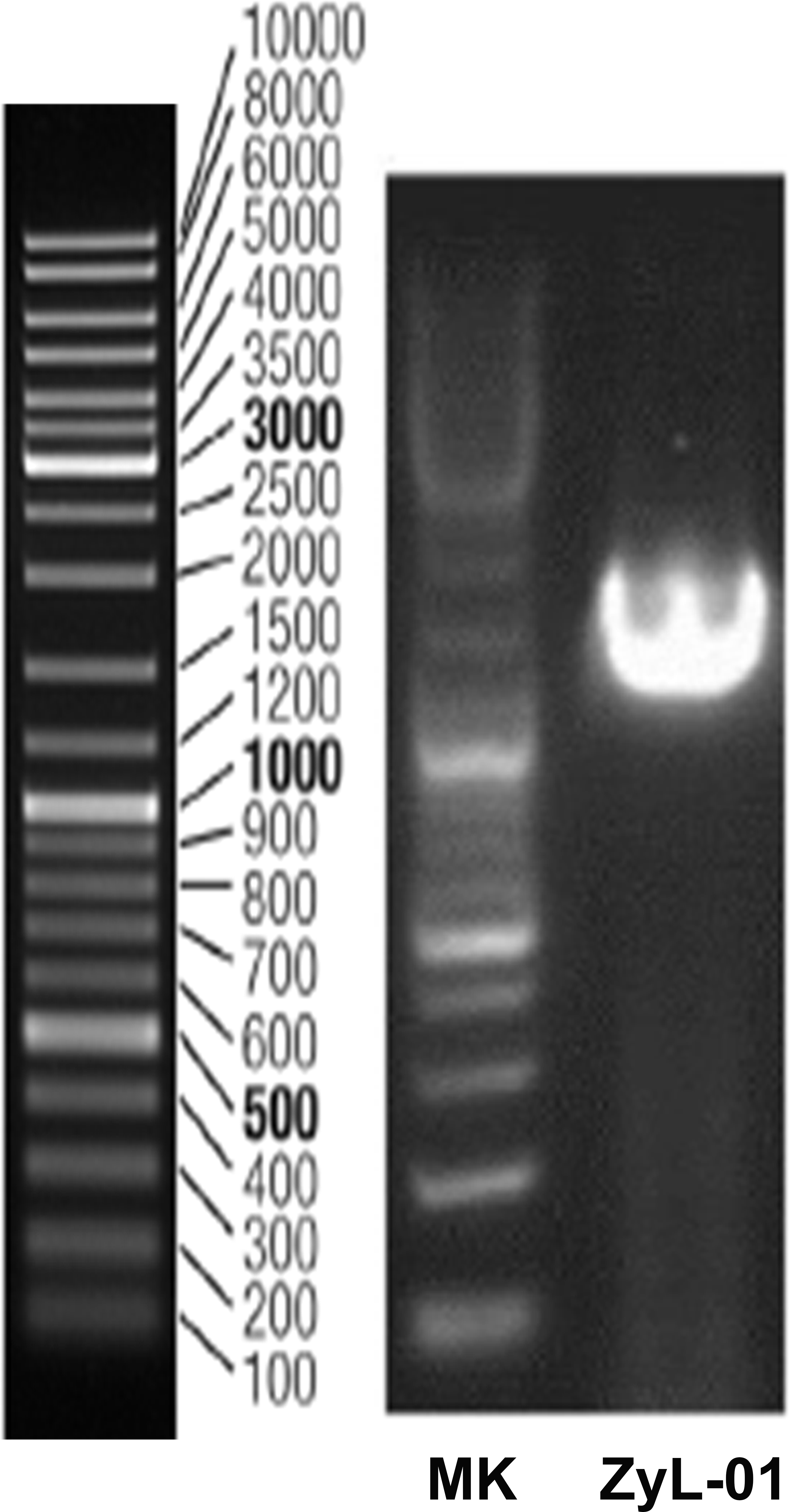

Supplement: Supplementary file 2 — Additional file 2: Fig S2. 16s rRNA gene sequencing analysis of the newly isolated strain ZyL-01. [file 13568_2020_1136_MOESM2_ESM.tif]

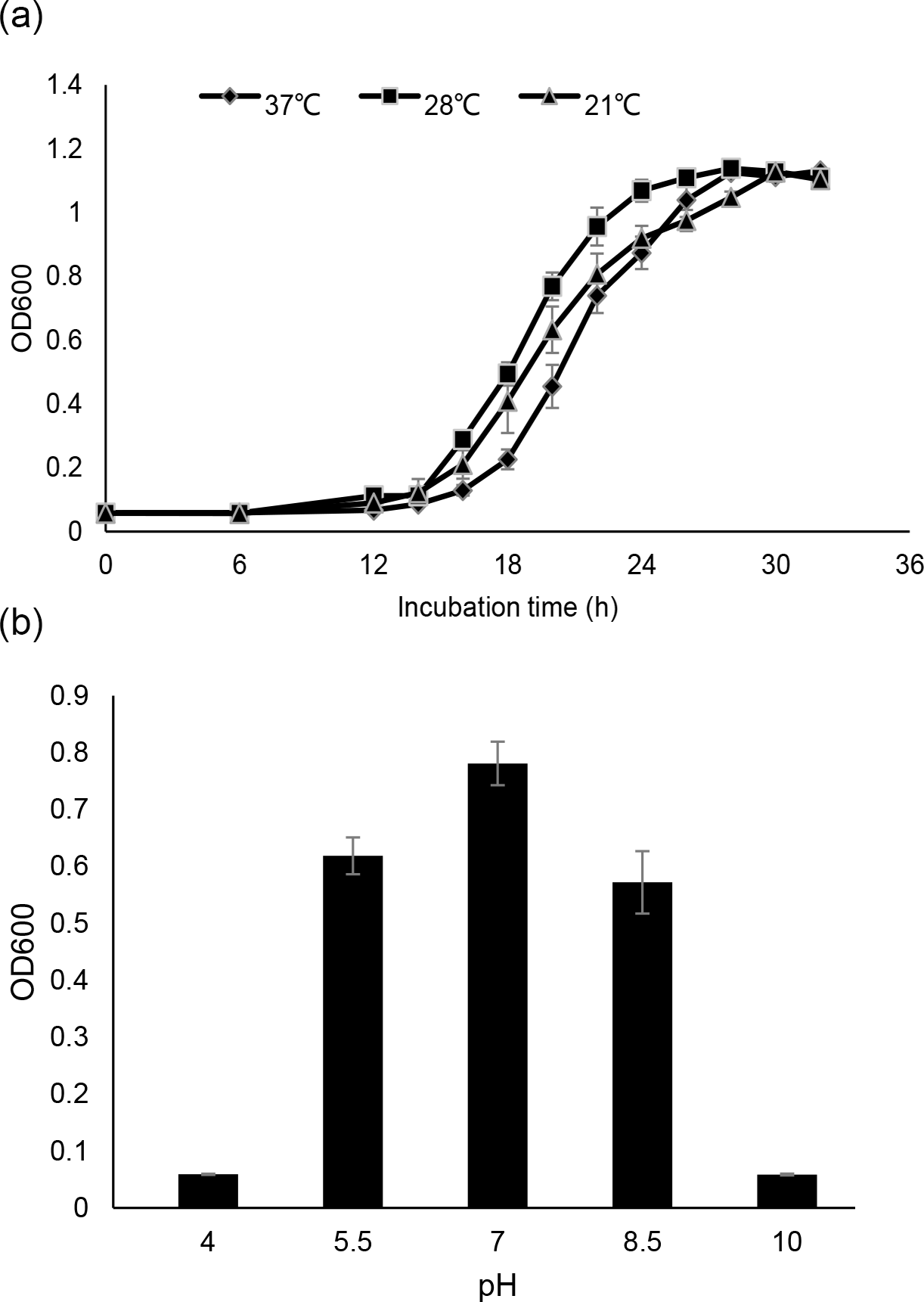

Supplement: Supplementary file 3 — Additional file 3: Fig S3. Growth characteristic of ZyL-01 with different temperatures (a) and pH values (b). [file 13568_2020_1136_MOESM3_ESM.tif]
