# Supplementary material for: Isolation and identification of a novel bacterium, Pseudomonas sp. ZyL-01, involved in the biodegradation of CL-20
Source: AMB Express. 2020 Oct 31;10:196. doi: 10.1186/s13568-020-01136-x (PMC7603440; doi:10.1186/s13568-020-01136-x)
Supplement: Supplementary file 4 — Additional file 4: Table S1. Numbers and percentages of unigenes in different databases. [file 13568_2020_1136_MOESM4_ESM.docx]

Table S1. Numbers and percentages of unigenes in different databases

| Database | Number of Unigenes | Percentage |
| --- | --- | --- |
| CDD | 5,505 | 86.77 |
| COG | 4,996 | 78.75 |
| NR | 6,300 | 99.31 |
| PFAM | 5,309 | 83.69 |
| Swissprot | 4,690 | 73.93 |
| TrEMBL | 6,294 | 99.21 |
| GO | 4,454 | 70.21 |
| KEGG | 2,797 | 44.09 |
| Annotated in at least one database | 6,303 | 99.35 |
| Annotated in all databases | 2,663 | 41.98 |
| Total Unigenes | 6,344 | 100.00 |
